# Endothelial Cell‐Specific Molecule‐1 (ESM1): An Endogenous Anticoagulant and Protective Factor in Venous Thrombosis

**DOI:** 10.1002/advs.202515994

**Published:** 2026-01-09

**Authors:** Changsheng Chen, Xiaojuan Ge, Dongxu Fu, Haijun Mei, Feng Lv, Chao Yang, Jiahao Lu, Xiaozhong Shen, Bowen Li, Xiaoning Wang, Dong Liu

**Affiliations:** ^1^ School of Life Sciences, Co‐innovation Center of Neuroregeneration Nantong University Nantong China; ^2^ Department of Interventional and Vascular Surgery Affiliated Hospital of Nantong University Nantong China; ^3^ Nantong Science and Technology College Nantong China; ^4^ Institute of Special Environmental Medicine Nantong University Nantong China; ^5^ Medical College of Nantong University Nantong China; ^6^ State Key Laboratory of Medical Genomics, Shanghai Institute of Hematology, National Research Center For Translational Medicine Ruijin Hospital affiliated to Shanghai Jiao Tong University School of Medicine Shanghai China; ^7^ Research Center of Clinical Medicine Affiliated Hospital of Nantong University Nantong China

**Keywords:** animal models, anticoagulation, biomarker, endothelial cell‐specific molecule‐1, venous thromboembolism

## Abstract

Deficiencies in endogenous anticoagulation pathways can lead to vascular occlusion and thrombosis. Endothelial cell‐specific molecule‐1 (ESM1), a proteoglycan secreted by endothelial cells, is elevated in patients with venous thromboembolism (VTE), yet its role in coagulation regulation remains undefined. Serum ESM1 concentrations are significantly higher in individuals with VTE (498.54 pg/mL) than in healthy controls (198.68 pg/mL), and the combination of ESM1 and D‐dimer increases diagnostic discrimination. The anticoagulant potential of ESM1 is assessed using time‐to‐occlusion (TTO) assays in zebrafish and mouse models, complemented by in vitro analyses of endogenous thrombin inhibitor activation. The anticoagulant effect of recombinant human ESM1 was further examined in mouse model. Loss of *esm1* in zebrafish results in vascular occlusion in the cardinal vein, whereas *esm1* overexpression dose‐dependently reduces venous thrombosis and prolongs TTO. Similarly, *Esm1* knockout in mice leads to an alteration of coagulation function, which is rescued by human ESM1 protein. Mechanistically, ESM1's anticoagulant function is found to rely on its covalently linked glycosaminoglycans (GAGs), which activate the thrombin inhibitor heparin cofactor II (HCII). This study uncovers a novel function of ESM1 in anticoagulation through HCII activation, highlighting its potential as a therapeutic target for preventing venous thrombus formation.

## Graphical abstract

This study identifies ESM1 as an endogenous anticoagulant that modulates venous thrombosis. It shows that ESM1 deficiency triggers vascular occlusion in zebrafish and mice, whereas its restoration or overexpression prolongs clot‐formation time. Mechanistic analyses reveal that ESM1 activates HCII through its dermatan‐sulfate chain, uncovering a previously unrecognized pathway for thrombin inhibition and a potential therapeutic target.

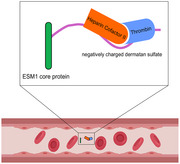

## 1 Introduction

Venous thromboembolism (VTE), a prevalent cardiovascular condition associated with significant morbidity and mortality, often remains asymptomatic, complicating clinical detection. Timely diagnosis and intervention are crucial for reducing VTE‐related fatalities [1]. The coagulation cascade, a critical pathway in blood clotting, is a prime target for reducing venous thrombosis in cardiovascular patients [2, 3]. This cascade triggers enzymatic reactions leading to thrombin production, which converts fibrinogen to fibrin, forming clots. Thrombin activity is naturally regulated by plasma proteins like antithrombin (AT) and heparin cofactor II (HCII), serpin family protease inhibitors that form stable complexes with thrombin [4]. These inhibitors form a stable, covalent 1:1 complex with thrombin, thereby neutralizing its activity [5]. The efficacy of thrombin inactivation by AT and HCII is significantly amplified in the presence of GAGs, a class of compounds that includes heparin, heparan sulfate (HS), and dermatan sulfate (DS) [6]. Heparin and its related compounds primarily enhance the anti‐coagulation process by binding to and activating AT, although they exhibit a lower affinity for HCII. DS stands out as a key anticoagulant, specifically interacting with HCII to augment thrombin inhibition [7]. GAGs are typically attached to proteoglycans (PGs), which are proteins comprising a core protein and one or more covalently linked GAG chains. The biological functions of PGs are largely dictated by the types and carbohydrate characteristics of their associated GAGs [8].

Endothelial cell‐specific molecule‐1 (ESM1), also known as endocan, is a DS‐containing proteoglycan predominantly produced by endothelial cells (ECs) [9]. Though primarily secreted by ECs, ESM1 expression has been identified in various cell types, including epithelial, cardiomyocytes, and chondrocytes [10, 11]. The protein core of ESM1 consists of 165 amino acids, with a DS side chain covalently linked to the 137th serine residue [12, 13]. ESM1 has been shown to interact with a broad spectrum of bioactive molecules, influencing key processes such as angiogenesis, cell proliferation, migration, and tumor growth [14, 15, 16, 17]. Clinical observations reveal a correlation between elevated serum ESM1 levels and multiple cardiovascular conditions, including atherosclerosis, coronary artery disease, and coronary artery ectasia [18, 19, 20, 21]. Our findings indicate higher ESM1 levels in the peripheral blood of VTE patients, hinting at a possible link between ESM1 and VTE pathogenesis. However, the exact role of this EC‐derived proteoglycan in the coagulation cascade and its potential as an endogenous thrombin inhibitor through HCII activation in venous thrombosis remains to be elucidated.

Despite the growing body of evidence implicating ESM1 in vascular biology, its role in coagulation remains largely unexplored, representing a significant gap in current knowledge. ESM1 is one of the few endothelial‐derived proteoglycans known to carry a DS chain that is structurally similar to DS moieties activating HCII, an established endogenous inhibitor of thrombin, thereby rendering ESM1 a biologically plausible regulator of anticoagulant pathways [4, 9]. Importantly, the functional anticoagulant activity of the DS chain on ESM1 has not been experimentally examined, leaving a critical gap between biochemical plausibility and physiological relevance. Consequently, the precise role of this endothelial‐derived proteoglycan in the coagulation cascade, and whether it contributes to endogenous anticoagulant mechanisms via HCII activation, remains unknown. Addressing this gap constitutes the central rationale for the present study, in which clinical analyses, zebrafish and mouse models, and biochemical assays are integrated to investigate how ESM1 influences thrombosis and coagulation in vivo.

This study employed the zebrafish model to investigate ESM1's role in coagulation and thrombosis. The transparent embryos allow high‐resolution, non‐invasive imaging of the circulatory system and in vivo processes. Genetic manipulation techniques like morpholinos and CRISPR/Cas9 facilitate disease model creation [22]. Additionally, zebrafish have conserved orthologs of mammalian blood coagulation factors and hemostatic pathways similar to humans [23, 24, 25]. Their high breeding capacity also makes them suitable for high‐throughput genetic and therapeutic screenings [26].

Consistent with prior studies, ESM1 is predominantly expressed in ECs as revealed by cross‐species comparative analyses. In situ hybridization assays further substantiate ESM1 expression in vascular tip cells. However, *esm1*‐knockout mutants show no defects in sprouting angiogenesis, contradicting the earlier view that ESM1 modulates tip cell behavior [14]. Surprisingly, Esm1 deficiency in zebrafish reduces blood flow and increases coagulation risk compared to wild‐type (WT) counterparts. Conversely, Esm1 overexpression restores anticoagulant capacity in knockouts and enhances it in WT. Similarly, *Esm1* knockout in mice reduces anticoagulant activity, which can be rescued by human ESM1 protein treatment. Mechanistically, ESM1's anticoagulant function is attributed to its DS side chain, which interacts with HCII to augment thrombin inhibition. Collectively, these findings position ESM1 as a significant modulator in the coagulation pathway, with promising clinical implications for therapeutic exploitation.

## 2 Results

### 2.1 ESM1 Levels are Positively Correlated with the D‐Dimer Levels in Patients with VTE

**FIGURE 1:**
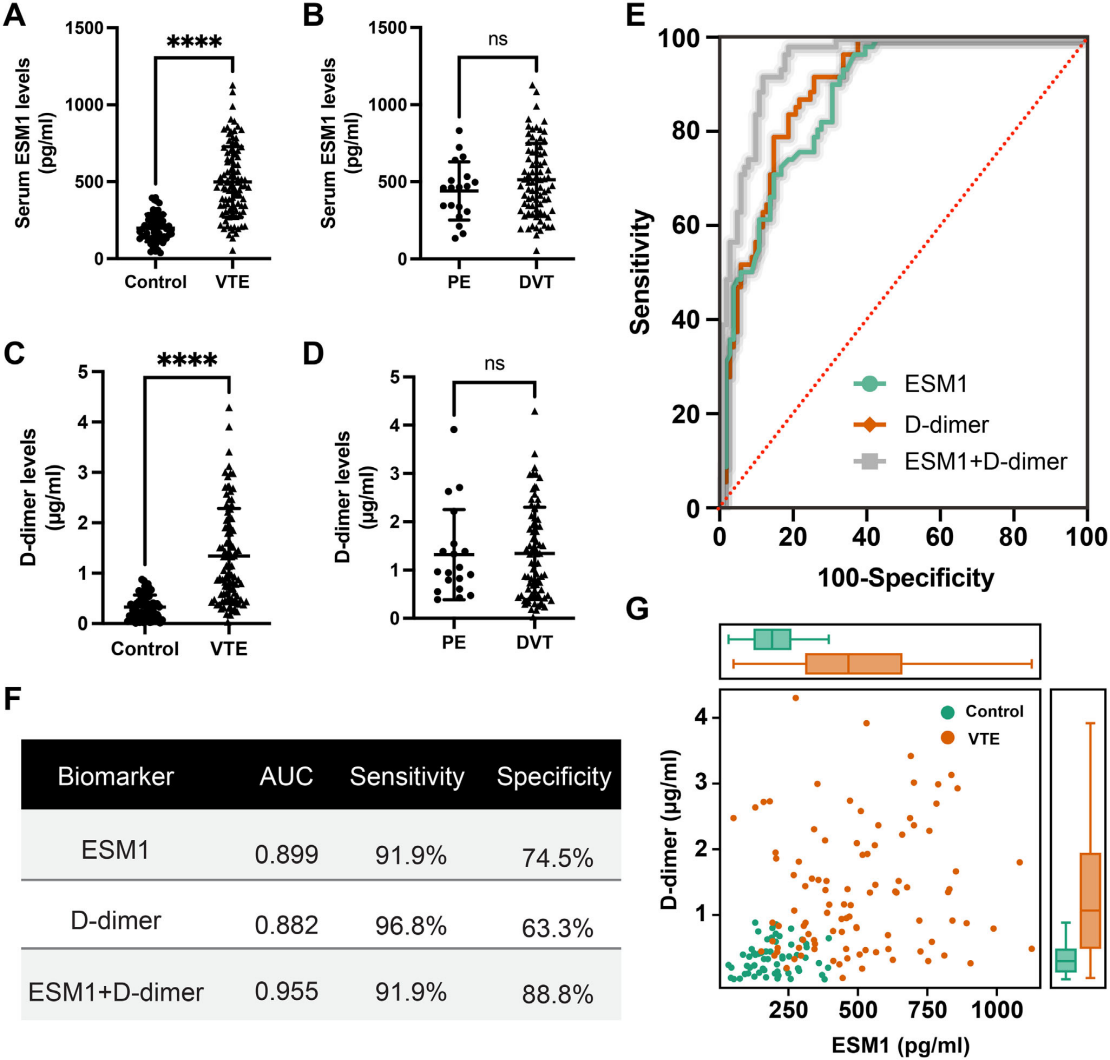
Serum ESM1 levels are elevated in venous thromboembolism (VTE) patients and positively correlated with the D‐dimer levels. (A and C) Concentrations of ESM1 in the serum (A) or D‐dimer in the plasma (C) from VTE patients and healthy subjects. (B and D) The comparison of serum ESM1 (B) or plasma D‐dimer (D) levels between deep vein thrombosis (DVT) and pulmonary embolism (PE) patients. (E and F) Receiver Operating Characteristic (ROC) curves (E) and analyses (F) for ESM1 and D‐dimer to identify venous thrombotic events. (G) The correlation between ESM1 and D‐dimer levels in VTE patients and healthy subjects.

This study enrolled 160 participants: 62 healthy controls (32 males, 30 females; mean age 64.35 ± 12.44 years) and 98 VTE patients (57 males, 41 females; mean age 65.31 ± 13.84 years). No significant differences in age or gender were observed between the groups (*p* = 0.5177 and *p* = 0.3337, respectively) (Table 1). Serum ESM1 levels were significantly elevated in VTE patients (498.54 ± 229.47 pg/mL) compared to controls (198.68 ± 89.62 pg/mL, *p* < 0.0001) (Figure 1A). High ESM1 levels were more prevalent in VTE patients (73.47%) than in controls (11.29%, *p* < 0.0001) (Table 2), indicating a strong positive correlation with VTE. ESM1 levels did not significantly differ with respect to age or sex (*p* = 0.6353 and *p* = 0.4270, respectively) (Table 3). Within the VTE group, 79 patients had deep vein thrombosis (DVT) and 19 had pulmonary embolism (PE), but no significant difference in ESM1 levels was found between DVT and PE patients (Figure 1B). The AUC for serum ESM1 in VTE diagnosis was 0.899 (95% CI: 0.854–0.945, *p* < 0.0001) (Figure 1E,F). At an optimal cut‐off value of 320.8 pg/mL, ESM1 demonstrated 91.9% sensitivity and 74.5% specificity (Figure 1E,F). ESM1's predictive capacity was comparable to D‐dimer (AUC 0.882, 95% CI: 0.832–0.931, *p* < 0.0001; optimal threshold 0.798 µg/mL, 96.8% sensitivity, 63.3% specificity) (Figure 1E,F). Plasma D‐dimer levels were also significantly higher in VTE patients than in controls, with no difference between PE and DVT patients (Figure 1C,D). Combining ESM1 and D‐dimer improved diagnostic accuracy (AUC 0.955, 91.9% sensitivity, 88.8% specificity). Elevated ESM1 levels often coincided with high D‐dimer levels (Figure 1G), suggesting ESM1's potential as a VTE biomarker. Overall, these results demonstrate that the serum ESM1 levels are positively correlated with the plasma D‐dimer levels in patients with VTE.

**TABLE 1:** Correlation of VTE with age and sex.

Group		number	VTE non‐VTE		Positive	P
Age	≤66^a^	72	42	30	58.33%	0.5177
	>66	88	56	32	63.64%	χ^2^=0.4692
Sex	Male	88	57	31	64.77%	0.3318
	Female	72	41	31	56.94%	χ^2^=1.022

^a^ Median age.

**TABLE 2:** Correlation of ESM1 level with VTE.

Group	number	ESM1 level		Positive	P
		low^a^ high^b^			
Control subjects	62	55	7	11.29%	<0.0001
VTE subjects	98	26	72	73.47%	χ^2^ = 58.74

^a)^ Serum ESM1 content lower than 320.8 pg/mL.

^b)^ Serum ESM1 content higher than 320.8 pg/mL.

**TABLE 3:** Correlation of ESM1 level with age and sex.

Group		number	ESM1 level		Positive	P
			low high			
Age	≤66	82	39	43	52.44%	0.6353
	>66	78	41	37	47.44%	χ^2^=0.4003
Sex	Male	88	47	41	46.59%	0.4270
	Female	72	33	39	54.17%	χ^2^=0.9091

### 2.2 ESM1 is Primarily Expressed in Microvascular ECs

**FIGURE 2:**
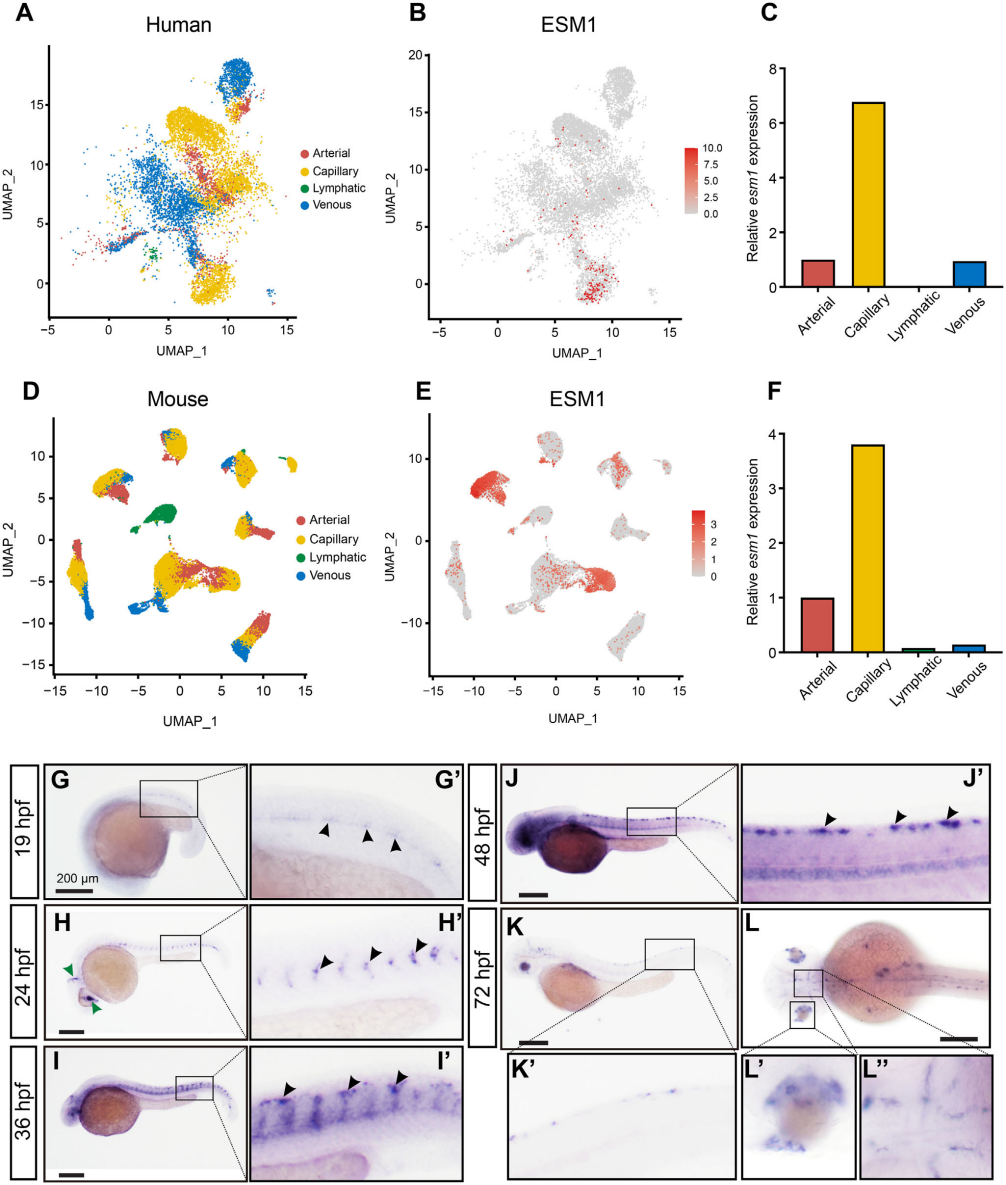
*ESM1* is highly expressed in microvascular endothelial cells (ECs). (A–F) Single‐cell transcriptomic profiling of human (A–C) and mouse (D–F) ECs from publicly available datasets [27, 28]. UMAP plots demonstrate that *ESM1* is primarily expressed in microvascular cells (capillary). (G–L) Whole‐mount in situ hybridized embryos showing the expressions of *esm1* is specifically expressed in zebrafish microvasculature.

Zebrafish possesses a single ortholog of the human *ESM1* gene. Multiple alignments of ESM1 protein sequences demonstrated high homology between zebrafish Esm1 and other vertebrate species (Figure S1). Similar to human ESM1, zebrafish Esm1 harbors a conserved O‐glycosylated serine site in its C‐terminal region. We investigated ESM1 expression by analyzing single‐cell RNA sequencing datasets from humans and mice [27, 28], revealing that ESM1 is predominantly expressed in microvascular ECs across species (Figure 2A–F). Using WISH, we confirmed that *esm1* is strongly expressed in zebrafish microvasculature, especially in intersegmental vessels (ISVs) and the dorsal longitudinal anastomotic vessel (DLAV) (Figure 2H–J). *Esm1* expression starts at 19 h post‐fertilization (hpf), peaks in ISVs at 36 hpf, and diminishes by 72 hpf (Figure 2G–K). These findings establish ESM1 as an endothelium‐specific gene primarily expressed in microvascular ECs across vertebrates.

### 2.3 Generation of *Esm1* Knockout Mutants

**FIGURE 3:**
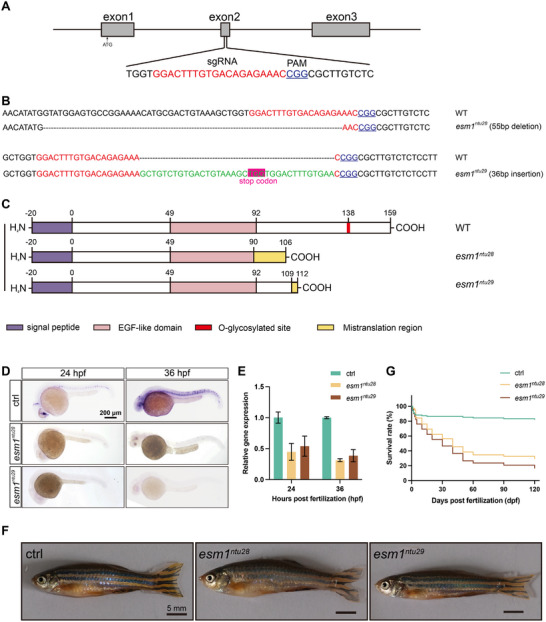
Generation of zebrafish *esm1* knock‐out using CRISPR/Cas9. (A) Schematic diagram showing gRNA targeting a site on the second exon of *esm1* gene. The start codon (ATG) site is indicated by the arrow. (B and C) Sequence alignments and schematics of Esm1 proteins encoded by WT, *esm1^ntu28^*, and *esm1^ntu29^* alleles. (D) Expression patterns of *esm1* transcripts detected by WISH in WT and mutants at 24 and 36 hpf. Scale bar, 200 µm. (E) Quantitative‐PCR analysis of *esm1* RNA levels in WT and mutants at 24 and 36 hpf. *Ef1a* was used as the housekeeping control. (F) Images of adult WT and mutants. Scale bar, 5 mm. (G) Survival rates of WT and mutants.

To generate *esm1* knockout zebrafish, the CRISPR/Cas9‐mediated genome editing technology was used. The second exon of zebrafish *esm1* was targeted with the aim of creating a frameshift and subsequent nonsense mutation prior to the O‐glycosylated site (Figure 3A). Two mutated alleles, *esm1^ntu28^* and *esm1^ntu29^*, carrying 55‐base pair deletions and 36‐base pair insertions in the second exon, respectively, were identified (Figure 3B). Both alleles caused frameshifts and premature stop codons, encoding truncated proteins that lacked the critical glycosylated site (Figure 3C). The mutations in *esm1* were validated by WISH and qRT‐PCR, and significant reductions of *esm1* transcripts were observed in both knockout mutants (Figure 3D,E). Notably, neither homozygous *esm1^ntu28/ntu28^* (hereafter referred to as *esm1^ntu28^*) nor *esm1^ntu29/ntu29^* (hereafter referred to as *esm1^ntu29^*) mutants exhibited any signs of developmental delay or growth restriction (Figure 3F). However, from 10 days post‐fertilization (dpf), homozygous mutants exhibited increased fragility and lethargy compared to their WT siblings. Furthermore, the survival rates of both *esm1^ntu28^* and *esm1^ntu29^* mutants progressively declined to less than 50% by 40 dpf (Figure 3G). In addition, mortalities in both mutants decrease between 40 to 60 dpf may result from the transition to sexual maturity and increase in aggressive behaviors (Figure 3G).

### 2.4 Loss‐of‐Function in *Esm1* has no Overt Effects on Sprouting Angiogenesis but Leads to Alterations in Coagulation Cascades

Rocha et al. has implied that Esm1 plays a crucial role in VEGF‐mediated sprouting angiogenesis [14]. In zebrafish, VEGF signaling is essential for the sprouting angiogenesis process, which involves the formation of ISVs from the dorsal aortae (DA) during development [29]. To further investigate the potential involvement of Esm1 in angiogenesis modulation, we conducted a study monitoring the development of ISVs in *Tg(fli1ep:EGFP‐CAAX)^ntu666^*;*esm1^ntu28^* and *Tg(fli1ep:EGFP‐CAAX)^ntu666^*;*esm1^ntu29^* embryos. The entire vasculature of these embryos was fluorescently labelled with GFP, allowing for precise visualization of angiogenic processes.

**FIGURE 4:**
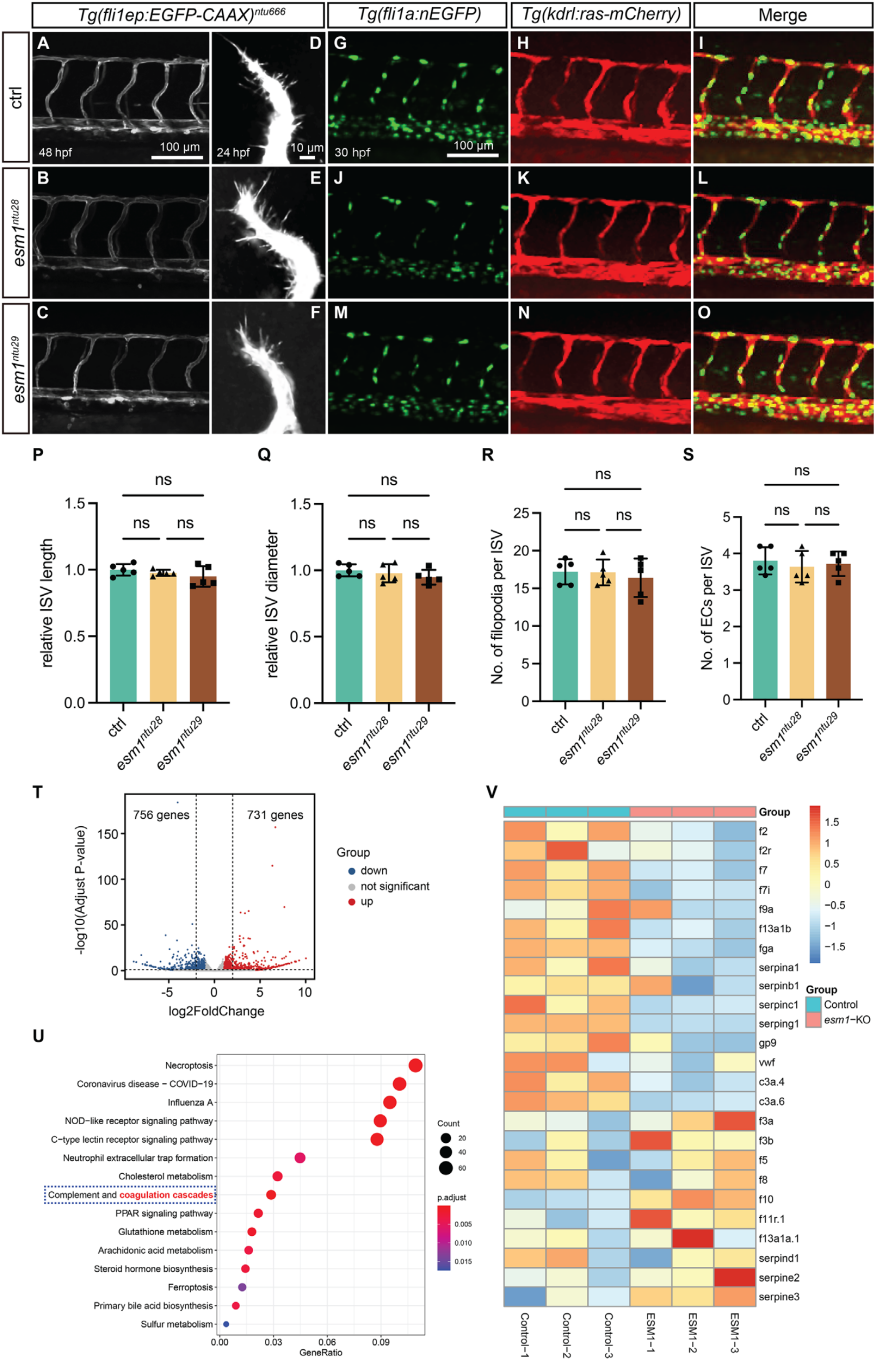
Loss‐of‐function in Esm1 has no overt effect on sprouting angiogenesis but leads to alterations in coagulation cascades. (A–C) Confocal images of ISV phenotypes in 48‐hpf *Tg(fli1ep:EGFP‐CAAX)^ntu666^* control embryos (*n=5*), *Tg(fli1ep:EGFP‐CAAX)^ntu666^*;*esm1^ntu28^* embryos (*n=5*), and *Tg(fli1ep:EGFP‐CAAX)^ntu666^*;*esm1^ntu29^* embryos (*n=5*). Scale bar, 100 µm. (D–F) Confocal images of filopodia of developing ISVs in 24‐hpf *Tg(fli1ep:EGFP‐CAAX)^ntu666^* control embryos (*n=5*), *Tg(fli1ep:EGFP‐CAAX)^ntu666^*;*esm1^ntu28^* embryos (*n=5*), and *Tg(fli1ep:EGFP‐CAAX)^ntu666^*;*esm1^ntu29^* embryos (*n=5*). Scale bar, 10 µm. (G–O) Confocal images of *Tg(fli1:nEGFP);Tg(kdrl:ras‐mCherry)* double transgenic embryos and *esm1* mutants at 30 hpf. Scale bar, 100 µm. (P and Q) Quantifications of ISV length and diameter in control and mutants at 48 hpf. (R) Quantification of filopodia number in developing ISVs in control and mutants at 24 hpf. (S) Quantification of EC number in ISVs in control and mutants at 30 hpf. Five embryos are analyzed for each group in **P‐S**. Data are shown as mean ± s.e.m. One‐way ANOVA analysis is applied. ns, not significant. (T−V) Whole‐genome transcriptomic profiling of 48‐hpf control embryos and *esm1^ntu28^* embryos. T, Volcano plot of downregulated genes (blue dots), upregulated genes (red dots), and unchanged genes (grey dots) in the *esm1*‐KO group versus the control group. (U) KEGG analysis of DEGs showing that the complement and coagulation cascades is enriched. (V) Heatmap based on bulk RNA‐seq data showing expression changes of the coagulation‐related DEGs.

Homozygous *esm1^ntu28^* and *esm1^ntu29^* mutants did not exhibit significant changes in trunk vascular development compared to controls (Figure 4A–C). The overall architecture of the trunk vasculature in *esm1* mutants at 48 hpf closely resembled their control siblings, with no significant differences in the length and diameter of mature ISVs between *esm1* mutants and wildtypes (Figure 4P,Q). To investigate whether there was a potential hypomorphic impact of truncated Esm1 products, we utilized a splicing morpholino targeting *esm1* to suppress its expression in *Tg(fli1ep:EGFP‐CAAX)^ntu666^* zebrafish embryos. The morphants exhibited vascular phenotypes analogous to those observed in *esm1* knockout mutants (Figure S2). Time‐lapse imaging revealed no impact on the process of ISV sprouting angiogenesis upon loss of *esm1* from 24 to 30 hpf (Figure S3).

Analysis of endothelial tip cell migration in 24 hpf embryos and stalk cell proliferation in 30 hpf embryos revealed no disruption of EC migration or proliferation in esm1 mutants (Figure 4D–O). Both the number of filopodia in primary angiogenic sprouts at 24 hpf and the EC counts per ISV remained intact in *esm1* mutants (Figure 4R,S), indicating that *esm1* deletion does not disrupt EC migration or proliferation during vascular development. Notably, embryos overexpressing *esm1* displayed a trunk vasculature pattern resembling wildtype, but differing from *vegfaa*‐expressing embryos, where ISVs exhibited excessive branching and aberrant, complex interconnections (Figure S4). RNA‐seq of 48‐hpf WT and *esm1^ntu28^* mutants revealed 731 upregulated and 756 downregulated genes (Figure 4T). Most angiogenesis‐ and vascular development‐related genes showed similar expression levels between esm1 mutants and controls (Figure S5). KEGG analysis indicated esm1 deletion enriched complement and coagulation cascades (Figure 4U), supported by RNA‐seq heatmap analysis showing significant alterations in coagulation‐related genes (Figure 4V). Collectively, these findings underscore the dispensability of zebrafish Esm1 for VEGF‐dependent sprouting angiogenesis and highlight its potential involvement in coagulation process.

### 2.5 Loss‐of‐Function in Esm1 Leads to the Decrease in the Blood Flow Velocity and the Formation of Blood Clots

**FIGURE 5:**
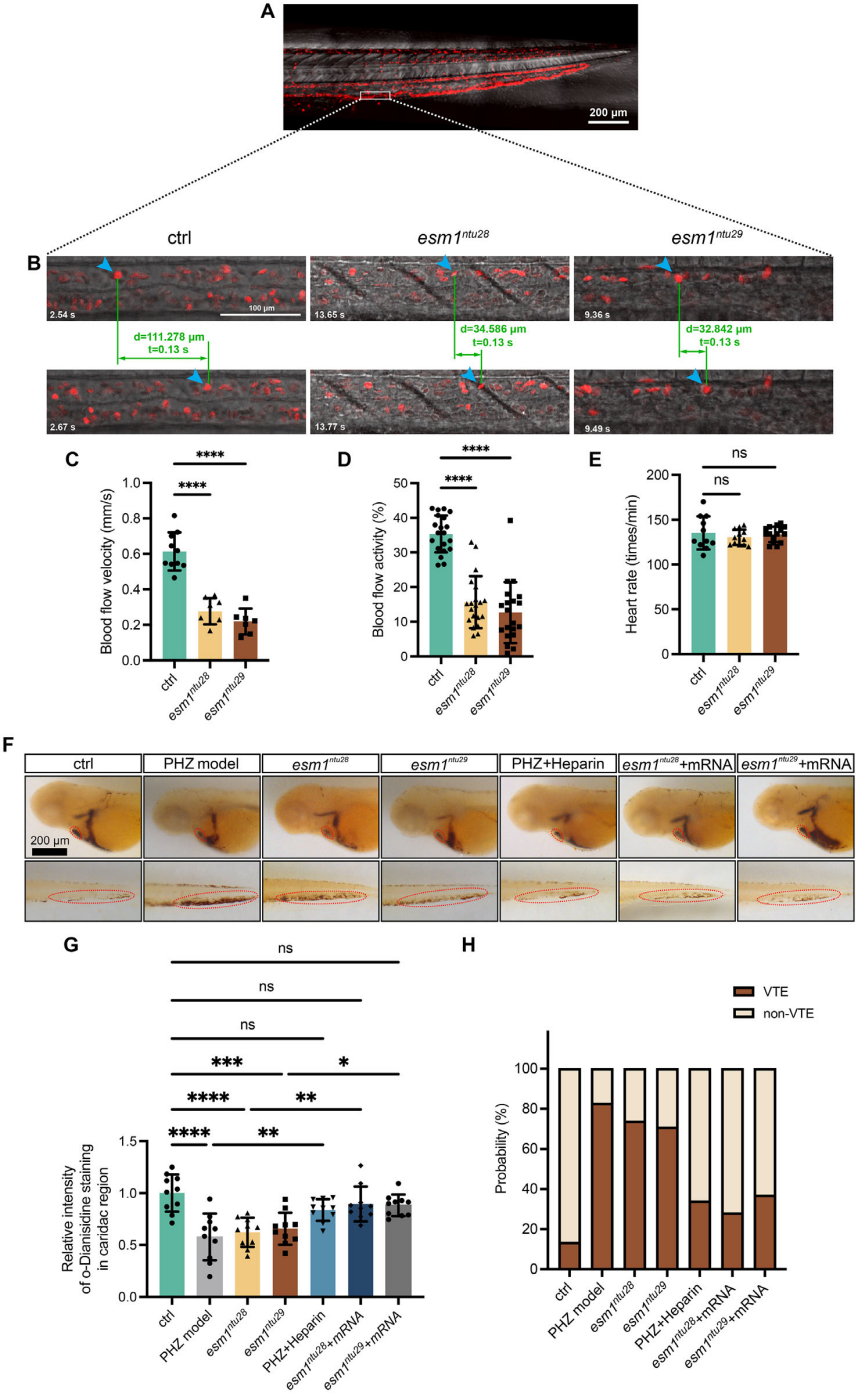
Esm1 loss‐of‐function induces thrombus formation. (A) Representative confocal images of red blood cells in *Tg(gata1:DsRed)* transgenic line at 48 hpf at a single Z‐stack layer. White boxed region indicates the representative position for blood flow quantification. Five different positions are selected for an individual fish. Scale bar, 200 µm. (B) Blood flow velocity is manually calculated by tracking red blood cells in two successive frames. (C) Comparison of blood flow velocity in control and mutants. (D, E) Blood flow activities and heartbeat rates in 48‐hpf control embryos and mutants are measured by DanioScope software. (F–J) Whole‐mount o‐dianisidine staining for hemoglobin at 48 hpf of control embryos, PHZ‐induced thrombotic models, *esm1*‐knockout embryos, heparin‐treated PHZ models, and *esm1*‐knockout embryos injected with *esm1* mRNA. Red dashed circles represent the cardiac or cardinal vein (CV). Scale bar, 200 µm. (G) Relative intensity of o‐Dianisidine staining in cardiac regions of 48‐hpf control embryos, PHZ‐induced thrombotic models, *esm1*‐knockout embryos, and *esm1*‐knockout embryos injected with *esm1* mRNA. (K) Incidence of thrombotic events (VTE) in 48‐hpf control embryos, PHZ‐induced thrombotic models, *esm1*‐knockout embryos, and *esm1*‐knockout embryos injected with *esm1* mRNA. Data are shown as mean ± s.e.m. One‐way ANOVA analysis is applied. ns, not significant. ^*^ *p* < 0.05, ^**^ *p* < 0.01, ^***^ *p* < 0.001, ^****^ *p* < 0.0001.

Despite the absence of morphological alterations in trunk vessels during vascular development in *esm1* mutants, we surprisingly discovered a reduction in blood flow velocity in these mutants (Figure 5A–D; Movies S1–S3). O‐dianisidine staining revealed decreased cardiac erythrocytes and increased erythrocyte accumulation in the caudal vein (CV) of mutants compared to controls (Figure 5F–H), resembling a zebrafish vein thrombosis model induced by phenylhydrazine (PHZ) treatment, an optimization of a previously published method [30]. These findings suggest that the loss of Esm1 function leads to vascular occlusion. Notably, while heartbeating initiated and persisted normally in *esm1* mutants (Figure 5E), and vascular formation was not hindered by the loss of Esm1, the reduced blood flow and erythrocyte aggregation in the CV suggest the development of thrombosis in the mutants. Rescue experiments with *esm1* mRNA injection into one‐cell‐stage mutants restored cardiac erythrocyte levels and reduced CV accumulation (Figure 5F–H). Similar results were observed in the PHZ model treated with heparin, confirming that Esm1 loss is responsible for vascular occlusion.

### 2.6 *Esm1* is Required and Sufficient for Anticoagulation

**FIGURE 6:**
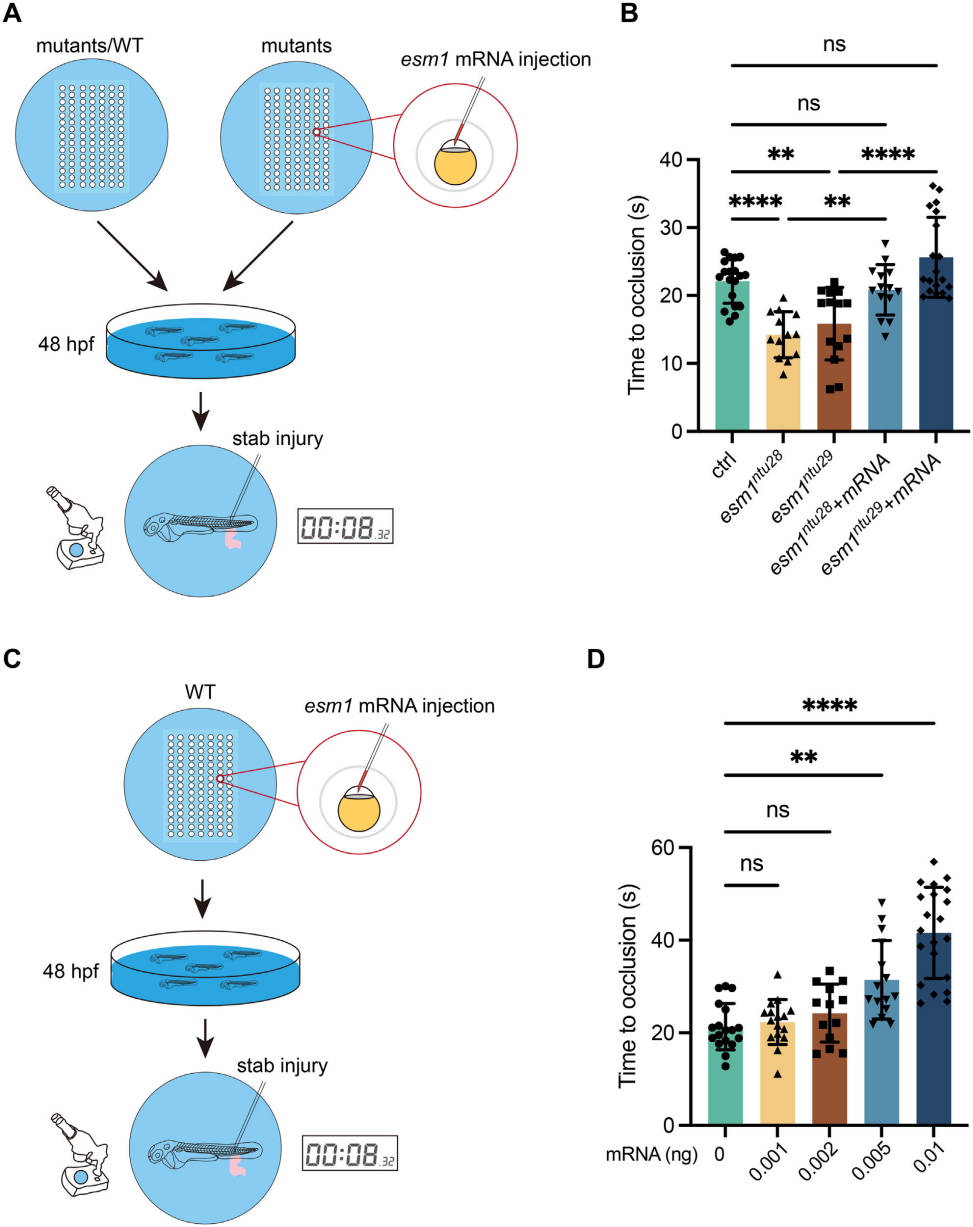
*Esm1* loss‐of‐function prolongs the time to occlusion (TTO). (A) Schematic representation of the experimental procedure for TTO measurement. (B) Comparisons of TTOs in control embryos, *esm1* mutants, and mutants injected with *esm1* mRNA. (C and D) Overexpressing *esm1* in control embryos leads to TTO prolongation. Data are shown as mean ± s.e.m. One‐way ANOVA analysis is applied. ns, not significant. ^**^ *p* < 0.01, ^****^ *p* < 0.0001.

We compared the TTO in the PCV of 48‐hpf WT, *esm1^ntu28^* and *esm1^ntu29^* embryos following injury. Specifically, we induced a stab injury by inserting a 0.1 mm stainless steel microcapillary needle into the end of CV (Figure 6A) and recorded the time elapsed from bleeding onset to occlusion. Unlike WT, both mutant strains exhibited a shorter TTO (Figure 6B). Notably, injecting *esm1* mRNA into the mutants led to a prolongation of the TTO (Figure 6B).

To further investigate the anticoagulant activity of Esm1, we injected *esm1* mRNA into WT zygotes and monitored their TTO after stab injury at 48 hpf. In comparison to embryos without mRNA injection, embryos receiving *esm1* mRNA injection displayed a prolonged TTO with a dose‐dependent effect (Figure 6C,D). These findings suggest that Esm1 plays a crucial role in anticoagulation in vivo, acting both sufficiently and necessarily.

### 2.7 Covalently‐Attached DS Glycosaminoglycan is Essential for the Anticoagulant Activity of *Esm1*

**FIGURE 7:**
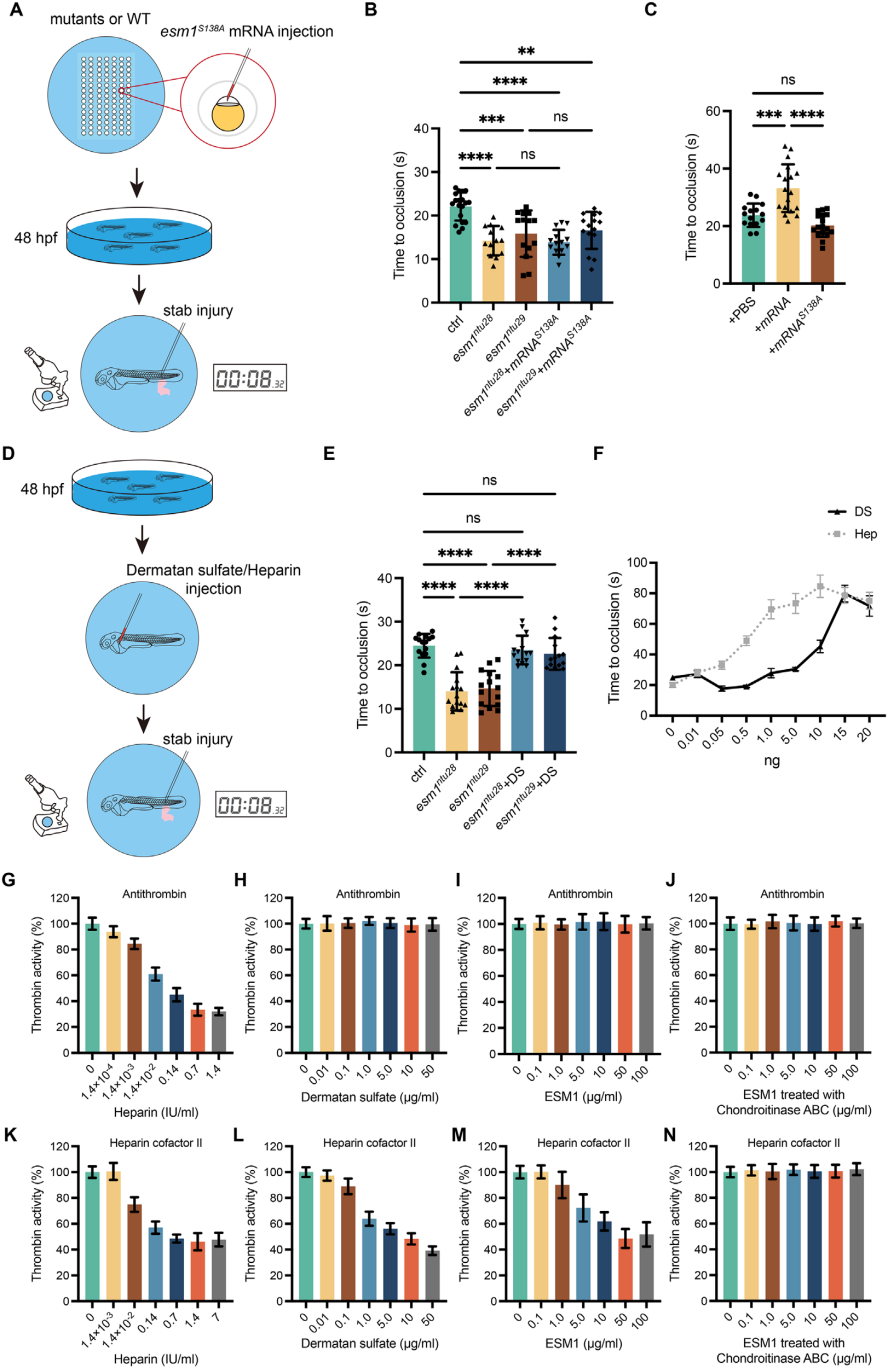
The covalently‐linked dermatan sulfate (DS) glycosaminoglycan of Esm1 is essential for its anticoagulant effect. (A–C) Expressing the point mutated Esm1 (Esm1^S138A^) has no effect on prolonging the TTO in zebrafish embryos after stab injury. (D) Dermatan sulfate or heparin is injected into 48‐hpf embryos for TTO measurement. (E, F) Injecting dermatan sulfate or heparin (1 IU ≈ 7 µg) prolongs TTOs in control embryos and *esm1* mutants. Data are shown as mean ± s.e.m. One‐way ANOVA analysis is applied. ns, not significant. ^****^ *p* < 0.0001. (G–O) Measurements of thrombin activity inhibition by heparin, dermatan sulfate, ESM1 protein, and Chondroitinase ABC‐treated ESM1 protein through activation of antithrombin (AT) (G–J) or heparin cofactor II (HCII) (L–O).

Esm1, a DS chain‐carrying proteoglycan, may function as an anticoagulant [31]. To verify if Esm1's anticoagulant activity arises from its DS side chain, we injected mutated *esm1* mRNA (*esm1^S138A^*) into one‐cell stage embryos of mutants and WT. This mRNA harbors a serine to alanine substitution at the 138th O‐glycosylated site. Post‐injection, we monitored the TTO following stab injury at 48 hpf (Figure 7A). Remarkably, injection of *esm1^S138A^* mRNA failed to rescue the shortened TTO in mutants or prolong it in WT (Figure 7B,C). However, injecting DS alone into 48‐hpf embryos’ CCV restored mutants’ TTO to wild‐type levels and dose‐dependently prolonged TTO in wild types (Figure 7D–F). Compared to heparin, DS showed weaker TTO‐prolonging effects at low concentrations but similar effects at high doses (Figure 7F).

Given DS inhibits thrombin by interacting with HCII [6], we explored if ESM1 exerts anticoagulant effects via HCII activation. In vitro assays showed human ESM1 protein inhibits thrombin activity with HCII, but not AT (Figure 7I,N). Heparin and DS also showed anticoagulant effects with HCII (Figure 7L,M), yet heparin displayed stronger thrombin inhibition with AT, while DS didn't affect thrombin activity in the presence of AT (Figure 7G,H). Notably, ESM1's thrombin inactivation capacity was eliminated when its DS chain was digested by chondroitinase ABC (Figure 7J,O).

**FIGURE 8:**
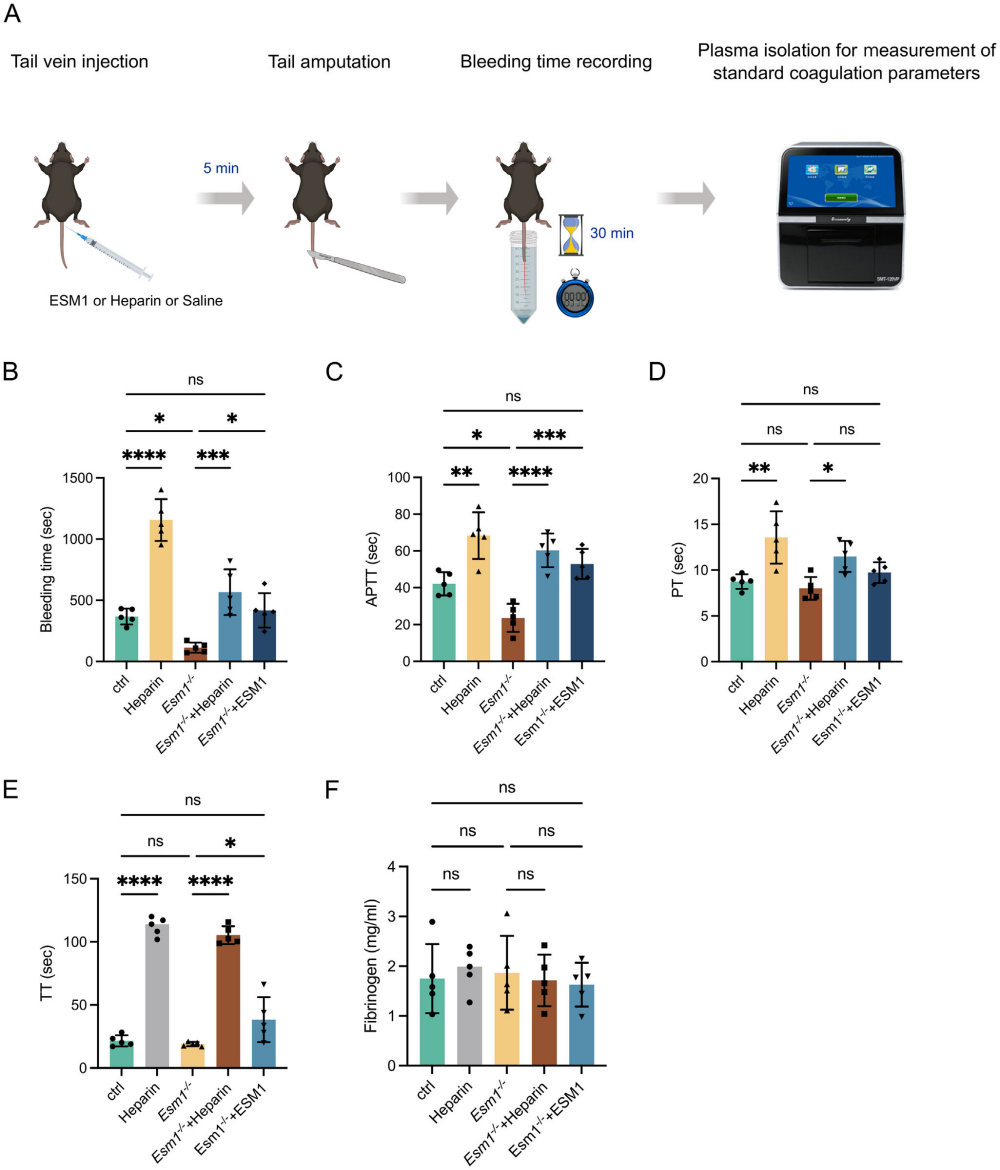
Loss of *Esm1* reduces the anticoagulant activity in mice and human ESM1 protein ameliorates the phenotype. (A) Schematic representation of the experimental procedure for tail vein bleeding time measurement and coagulation assessments in mice. (B‐D) Comparisons of bleeding time (B), APTT (C), and PT (D) in controls, heparin‐injected mice, *Esm1*‐KO mutants, and *Esm1*‐KO mutants injected with Heparin or recombinant human ESM1 protein. Data are shown as mean ± s.e.m. One‐way ANOVA analysis is applied. ns, not significant. ^*^ *p* < 0.05, ^**^ *p* < 0.01, ^***^ *p* < 0.001, ^****^ *p* < 0.0001.

We further investigated the protective role of ESM1 against blood coagulation using *Esm1*‐knockout (*Esm1^ko/ko^*) mice (Figure 8A). Compared to controls, *Esm1^ko/ko^* mice exhibited shorter bleeding times and reduced activated partial thromboplastin time (APTT) and prothrombin time (PT) (Figure 8B–D), whereas thrombin time (TT) remained unchanged (Figure 8E). Administration of low‐dose heparin (50 IU/kg) increased bleeding time, APTT, PT, and TT in both groups (Figure 8B–E). Injection of recombinant human ESM1 protein (1 µg/mouse) into *Esm1^ko/ko^* mice prolonged bleeding time, APTT, PT, and TT to levels comparable with control mice (Figure 8B–E). However, the plasma concentration of fibrinogen remained unchanged in all groups (Figure 8F).

Collectively, these findings suggest that ESM1 protein has a pharmacological effect in inhibiting coagulation. Specifically, ESM1 exerts its anticoagulant effect through its covalently linked DS chain in a way activating the plasma serpin protease inhibitor HCII.

## 3 Discussion

VTE, a leading cause of vascular mortality globally, poses a significant public health challenge due to its associated morbidity and mortality [32]. Accurate diagnosis and early treatment are crucial for effective thromboprophylaxis. Heparin represents one of the most frequently used anticoagulants in preventing VTE. It belongs to the class of GAGs, which are linear polymers of disaccharides with variable lengths that are modified by sulfation and/or (de)acetylation to a variable extent [33, 34]. Besides heparin, there are another five types of GAGs, including heparan sulfate, chondroitin sulfate, dermatan sulfate, keratan sulfate, and hyaluronan [34]. GAGs are typically covalently linked to core proteins, forming PGs that play vital roles in various biological activities and physiological processes, such as extracellular matrix assembly, cell behavior modulation, coagulation control, inflammation, lipid metabolism, and wound healing [8, 35].

ESM1, also known as endocan, is an endothelium‐derived DSPG first identified in human umbilical vein endothelial cells [9]. Our study found significantly elevated serum ESM1 levels in VTE patients compared to controls, suggesting its potential as a VTE biomarker. While plasma D‐dimer is a widely used biomarker for the diagnosis of VTE, its clinical utility is limited by its lack of specificity since elevated D‐dimer levels are common in various conditions, including cancer, pregnancy, surgery and trauma [36]. Consequently, D‐dimer level alone is insufficient to confirm a VTE diagnosis. Our analysis revealed that the combination of ESM1 and D‐dimers significantly improved diagnostic accuracy. Further investigation with a larger patient cohort in a blinded, prospective study is needed to refine the sensitivity, specificity, and clinical utility of this combined biomarker approach. Our findings highlight the potential of circulating ESM1 as a complementary marker in VTE assessment.

In light of these findings, our study also raises the possibility that circulating ESM1 may hold translational value as a complementary biomarker in VTE assessment. Unlike D‐dimer, which reflects fibrin degradation and is frequently elevated in numerous non‐thrombotic conditions [36], ESM1 originates from ECs and may more directly report endothelial activation or glycocalyx perturbation during thrombus formation. This mechanistic distinction suggests that combining ESM1 with D‐dimer could enhance diagnostic specificity, particularly in clinical settings where D‐dimer alone yields a high false‐positive rate, such as inflammation, malignancy, or postoperative states. Moreover, because ESM1 is linked to endogenous HCII‐mediated anticoagulant capacity, its measurement may provide insight into the balance between pro‐thrombotic and compensatory anticoagulant responses. Although further validation in larger prospective cohorts is needed, these considerations support the potential utility of ESM1 as an adjunct biomarker to improve VTE risk stratification and early diagnostic accuracy.

Research conducted in zebrafish demonstrated that genetic deletion of *esm1* results in sluggish blood flow and erythrocyte aggregation in the cardinal vein, suggestive of venous thrombosis formation. This phenotype was consistent in both *esm1*‐knockout mutants. Both *esm1^−/−^* genotypes exhibited accelerated mortality from one month post fertilization, potentially associated with the transition to sexual maturity and increase in aggressive behaviors. Interestingly, overexpressing *esm1* via mRNA injection in mutants restored blood flow, prevented vascular occlusion, and prolonged TTO. Furthermore, overexpressing *esm1* in WT also extended TTO. These findings highlight a novel function of ESM1 in inhibiting blood coagulation. However, this seems contradictory to the fact that ESM1 levels are higher in VTE patients. This increase in ESM1 might be the body's compensatory response to vein thrombosis. Additionally, ESM1 is a component of the endothelial glycocalyx, a carbohydrate‐rich layer lining the luminal surface of the vascular endothelium [37]. Membrane‐bound and soluble proteoglycans and glycoproteins are embedded within or layered on this network to form a mesh‐like structure [34]. The elevated ESM1 levels in VTE patients may result from the disruption or shedding of the glycocalyx under this pathological condition [38].

While ESM1 is known as an endothelial tip cell marker crucial for sprouting angiogenesis [14], our study found no impairment in sprouting angiogenesis in Esm1‐deficient zebrafish, contradicting previous mouse studies. This discrepancy might stem from differences in research models. Tamoxifen‐inducible Cre recombinase estrogen (CreER) system in angiogenesis assays may introduce toxic effects that independently inhibit retinal angiogenesis, potentially interfering with the interpretation of such studies [39]. As CreER‐mediated gene targeting remains a key method for angiogenesis research, potential CreER toxicity should be considered when performing such study and appropriate controls should be included. Furthermore, although *Esm1* knockout mice display shortened tail bleeding time, APTT, and PT, these changes indicate a disturbance in coagulation rather than definitive anticoagulant deficiency. A more complete evaluation of their coagulation pathways and testing across diverse thrombosis models will be necessary to clarify the precise role of ESM1 in thrombotic regulation.

Additionally, ESM1 level has been reported to be associated with a myriad of cardiovascular‐related diseases, including atherosclerosis (AS) [40], coronary artery disease (CAD) [41], hypertension (HT) [19], diabetes mellitus (DM) [42]. Elevated serum ESM1 levels are commonly observed in these diseases and often correlate with disease severity. Although our data consistently show that full‐length ESM1 exerts an anticoagulant effect via activation of HCII in vivo and in vitro, we cannot exclude the possibility that the immunoreactive material detected in patient serum is functionally heterogeneous. A proteolytic fragment of ∼14 kDa (p14) generated by cathepsin‐G cleavage has been described in human plasma [43]. The anticoagulant properties of this fragment remain unknown; however, its core protein may retain the capacity to interact with HCII and function as a biologic competitor of full‐length ESM1 [44]. We therefore propose that the observed paradox—elevated total ESM1 immunoreactivity despite ongoing thrombotic risk—may reflect accumulation of p14 (or other cleaved species) that competes with the full‐length DS‐bearing proteoglycan for HCII binding, thereby functionally antagonizing the anticoagulant response. Future studies characterizing circulating ESM1 isoforms, comparing their HCII‐binding affinities, and assessing coagulation function using size‐fractionated patient plasma will be essential to evaluate this mechanism.

Our study reveals ESM1's novel anticoagulant role in antithrombotic regulation, suggesting elevated ESM1 in thrombosis patients may be a compensatory mechanism. While the precise role of circulating ESM1 in thrombogenesis remains incompletely understood, it is hypothesized to play a key role in antithrombotic regulation, both during prothrombotic and post‐thrombotic states. From a clinical perspective, circulating ESM1 may serve as a biomarker for predicting cardiovascular risks and itself can be a pleiotropic target of cardiovascular drugs.

## 4 Experimental Section

### 4.1 Study Approval

Experiments using human samples were approved by the Affiliated Hospital of Nantong University (2020‐K013). The study involved 98 consecutive VTE patients (2021‐2023) and 62 healthy controls. All participants provided informed written consent following Declaration of Helsinki principles.

### 4.2 Baseline Clinical Data Collection

Baseline clinical characteristics were extracted from electronic medical records blinded to ESM1 measurements. The following variables were collected: (i) the interval between onset of the first symptomatic event and diagnostic imaging; (ii) the interval between objective confirmation of VTE and blood sampling; (iii) presenting symptoms, classified as DVT or PE; and (iv) anticoagulation status at the time of sampling (none, prophylactic dose, or therapeutic dose initiated after imaging). The median time from symptom onset to diagnostic imaging was 3 days (IQR, 1–5), and blood sampling occurred a median of 1 day (IQR, 0–2) after confirmation of VTE. Among the 98 included cases, 80.6% (79/98) presented with DVT and 19.4% (19/98) with PE. At the time of sampling, 31 patients (31.6%) were receiving prophylactic‐dose anticoagulation, and 12 (12.2%) had initiated therapeutic anticoagulation after imaging but prior to blood draw. Patients receiving therapeutic anticoagulation for >24 h before blood sampling were excluded from the biomarker analysis.

### 4.3 Animals

The wild‐type AB line and transgenic lines *Tg(fli1ep:EGFP‐CAAX)^ntu666^* [45], *Tg(gata1:DsRed)* [46], and *Tg(fli1a:nEGFP::kdrl:ras‐mCherry)* [22] were used in this study. The C57BL/6J mice and *Esm1^ko/ko^* mice used in this study were purchased from GemPharmatech Co. Ltd. (Jiangsu, China). Animal procedures followed NIH Guidelines and were approved by Nantong University's Laboratory Animal Center (S20200408‐025). Best efforts were made to minimize the number of animals used and prevent their suffering.

### 4.4 Blood Collection and Measurements

Blood samples were collected pre‐treatment from VTE patients and controls, allowed to clot for 30 min, then centrifuged at 3000 g for 10 min. Serum was stored at ‐80°C. Human ESM1 levels were measured via ELISA (E‐EL‐H1557, Elabscience) with a detection range of 15.63‐1000 pg/mL. For D‐dimer assay, blood samples were collected into sodium citrate tubes and centrifuged at 2,500 × g for 15 min to obtain platelet‐poor plasma. Plasma samples were stored at ‐80°C until analysis. Plasma D‐dimer levels were assessed using INNOVANCE D‑dimer reagent on automated coagulation analyzers (Sysmex).

### 4.5 Whole‐Mount In Situ Hybridization (WISH)

WISH with antisense RNA probes was performed as described previously [47]. The template for detecting esm1 (NM_001076741.1) was amplified from cDNA with forward primer 5′‐TTTTGGAGAGACTGAGGCGT‐3′ and reverse primer 5′‐TGCTTTCAGTGTTGGTGTCG‐3′. Embryo images were acquired using an Olympus MVX10 stereomicroscope with a DP71 camera.

### 4.6 Generation of *Esm1* Knock‐Out Mutants

To generate *esm1* knock‐out mutant zebrafish, we used a CRISPR/Cas9‐mediated approach. CRISPR/Cas9 target sites were designed to identify the sequences in the second exon of *esm1* gene. Two mutants with 55 bp deletion and 36 bp insertion at the second exon, causing premature stop codons, were identified. F0 founders were crossed with transgenic lines to generate heterozygotes. All the homozygous mutants used were derived from e*sm1^+/−^* incrosses.

### 4.7 Blood Flow Analysis

Blood flow in the cardinal vein of zebrafish was analyzed via two methods. First, manual tracking of red blood cells in *Tg(gata1:DsRed)* zebrafish involved calculating velocity as movement distance divided by time between two frames, with five cells measured per fish and five to ten fish per group. Second, Danioscope's image analysis algorithms measured flow by analyzing pixel changes in selected CV parts, with ten fish per group and five locations per fish. Results were presented as activity percentages.

### 4.8 O‐Dianisidine Staining for Erythrocytes

Erythrocyte levels were assessed via o‐dianisidine staining, as previously described [48]. Briefly, the dechorionated embryos were stained for 15 min with a solution consisting of o‐dianisidine (0.6 mg/mL), sodium acetate (0.01 mol/L), 0.65% hydrogen peroxide, and 40% (v/v) ethanol in the dark.

### 4.9 Time To Occlusion (TTO) Measurement

TTO analysis was performed to evaluate anticoagulant capacity in 48‐hpf zebrafish embryos following stab injury at the caudal vein, with modifications from ref. [49]. To examine the effects of *esm1* mRNA on anticoagulation, different concentrations of mRNA was injected into one‐cell stage embryos and the TTO was measured at 48 hpf. For dermatan sulfate (Sigma‐Aldrich) or heparin (J&K Scientific), it was injected into the common cardinal veins (CCV) of 48‐hpf embryos and the TTO was measured after 10 min.

### 4.10 Mice Anticoagulation Measurement

Mice at 8‐12 weeks of age were used in this study. Mice tail‐bleeding time was measured as described previously [50]. Mice were anesthetized with 2% isoflurane in 30% O_2_/70% N_2_ at 1 L/min, then placed horizontally on a tail clip platform. Tails were amputated 2 mm from the tip and immersed in 37°C saline. Bleeding was monitored for 30 min, summing intermittent bleeding times. Post‐experiment, blood was collected from the orbital vein in trisodium citrate, plasma obtained via 5000 g centrifugation for 10 min, and stored at ‐80°C. Standard coagulation parameters, including prothrombin time (PT), activated partial thromboplastin time (APTT), thrombin time (TT), and plasma fibrinogen levels were measured using the V7 coagulation test kit (BIOSTAR) via Vet Chemistry Analyzer SMT‐120VP (Seamaty Technology). For therapeutic agents, they were injected intravenously into the lateral tail vein 5 min before amputation.

### 4.11 Anti‐Thrombin Activity Measurement

The anti‐thrombin activities were measured as described previously [51]. Reactants included 0.1 IU/mL AT (Adhoc International Technologies) or 68 nM HCII (Thermo Fisher Scientific), 1 IU/mL thrombin (Adhoc International Technologies), and varying amounts of recombinant ESM1 protein (Sino Biologicals), DS (Sigma‐Aldrich), heparin (Sigma‐Aldrich), or chondroitinase ABC (Sigma‐Aldrich)‐treated ESM1. In 96‐well plates, 120 µL mixtures were incubated at 37°C for 2 min, then 40 µL thrombin was added. After 5 min, 40 µL of 1 mM thrombin chromogenic substrate S‐2238 was added, and absorbance at 405 nm was recorded after 20 min. The 405 nm absorbance indicated residual thrombin activity.

### 4.12 Statistical Analysis

Statistical analysis used two‐tailed, unpaired Student's t‐test, One‐way ANOVA, Chi‐square test, and/or Fisher's exact test as described in figure legends. The chi‐square test (χ2‐value) compared categorical data between groups. Data are presented as mean ± s.e.m., with P < 0.05 considered statistically significant. Receiver–operator characteristic (ROC) curves evaluated biomarker diagnostic accuracy for VTE by analyzing sensitivity and specificity across biomarker concentrations. AUC comparative analysis identified the most accurate biomarkers. ROC curves for combined biomarkers and correlation analysis were generated by CombiROC [52] and OmicShare Tools [53].

## Author Contributions

D.L., C.C., and X.W. supervised and designed this project. C.C. wrote the manuscript. C.C., X.G., X.S., B.L., and D.F. analyzed the data. X.G., D.F., F.L., C.Y., J.L., and X.W. performed the experiments; H.M. collected the clinical data. D.L. and C.C. revised the manuscript.

## Conflicts of Interest

The authors declare no conflicts of interest.

## Supporting information




**Supporting File 1**: advs73730‐sup‐0001‐SuppMat.docx.

**Supporting File 2**: advs73730‐sup‐0002‐MovieS1.mp4.

**Supporting File 3**: advs73730‐sup‐0003‐MovieS2.mp4.

**Supporting File 4**: advs73730‐sup‐0004‐MovieS3.mp4.

## Data Availability

The data that support the findings of this study are available on request from the corresponding author. The data are not publicly available due to privacy or ethical restrictions.
