# Supplementary material for: Endothelial Cell‐Specific Molecule‐1 (ESM1): An Endogenous Anticoagulant and Protective Factor in Venous Thrombosis
Source: Adv Sci (Weinh). 2026 Jan 9;13(16):e15994. doi: 10.1002/advs.202515994 (PMC13042502; doi:10.1002/advs.202515994)
Supplement: Supplementary file 1 — Supporting File 1: advs73730‐sup‐0001‐SuppMat.docx. [file ADVS-13-e15994-s002.docx]

Supporting Information

Title: Endothelial Cell-Specific Molecule-1 (ESM1): An Endogenous Anticoagulant and Protective Factor in Venous Thrombosis

*Changsheng Chen^1,*^ , Xiaojuan Ge^1^, Dongxu Fu^2^, Haijun Mei^3^, Feng Lv^4^, Chao Yang^5^, Jiahao Lu^1^, Xiaozhong Shen^6,7^, Bowen Li^1^, Xiaoning Wang^8, *^, Dong Liu^1,2, *^*


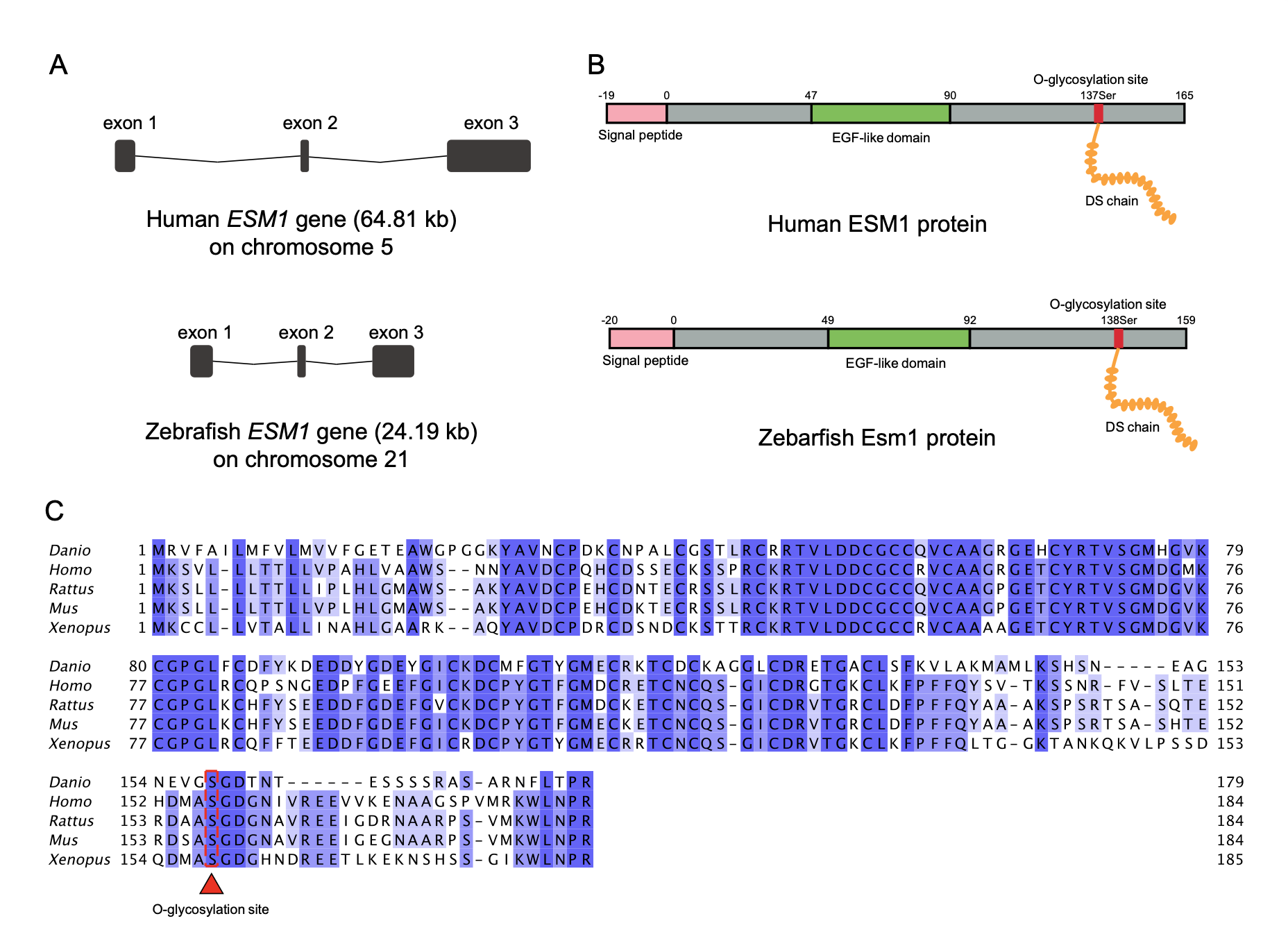


**Supplementary Figure 1. ESM1 is highly conserved in vertebrates. A and B,** Schematic representation of ESM1 gene and protein structures. **C,** Alignment of ESM1 protein sequence in in zebrafish, human, rat, mouse, and frog. The multiple sequence alignment result is produced by T-coffee and modified with JalView software.


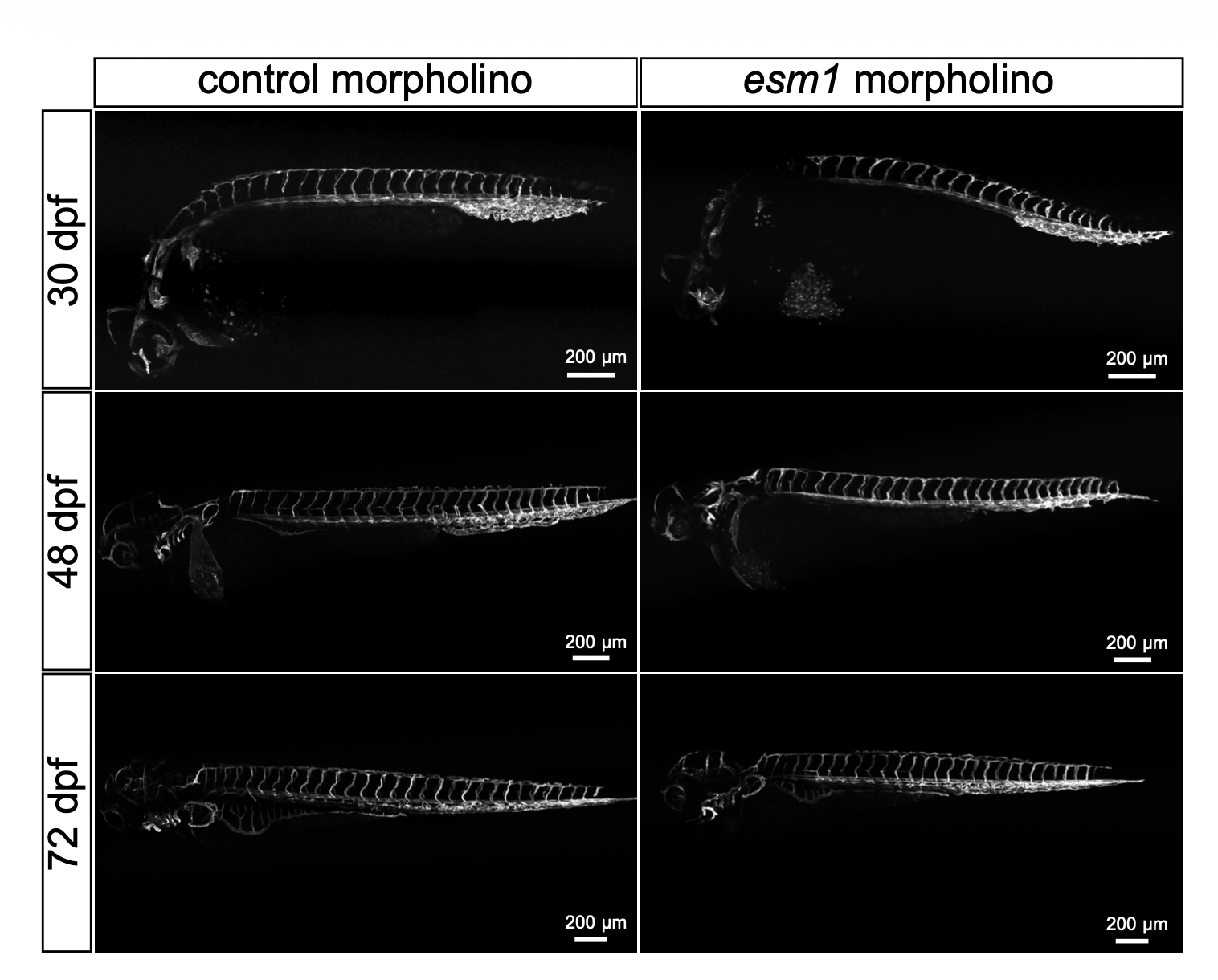


**Supplementary Figure 2.** Confocal images of the vascular system in *Tg(fli1ep:EGFP-CAAX)^ntu666^* control embryos and esm1 morpholino-injected *Tg(fli1ep:EGFP-CAAX)^ntu666^* embryos at 30, 48, and 72 hpf. Scale bars, 200 μm.


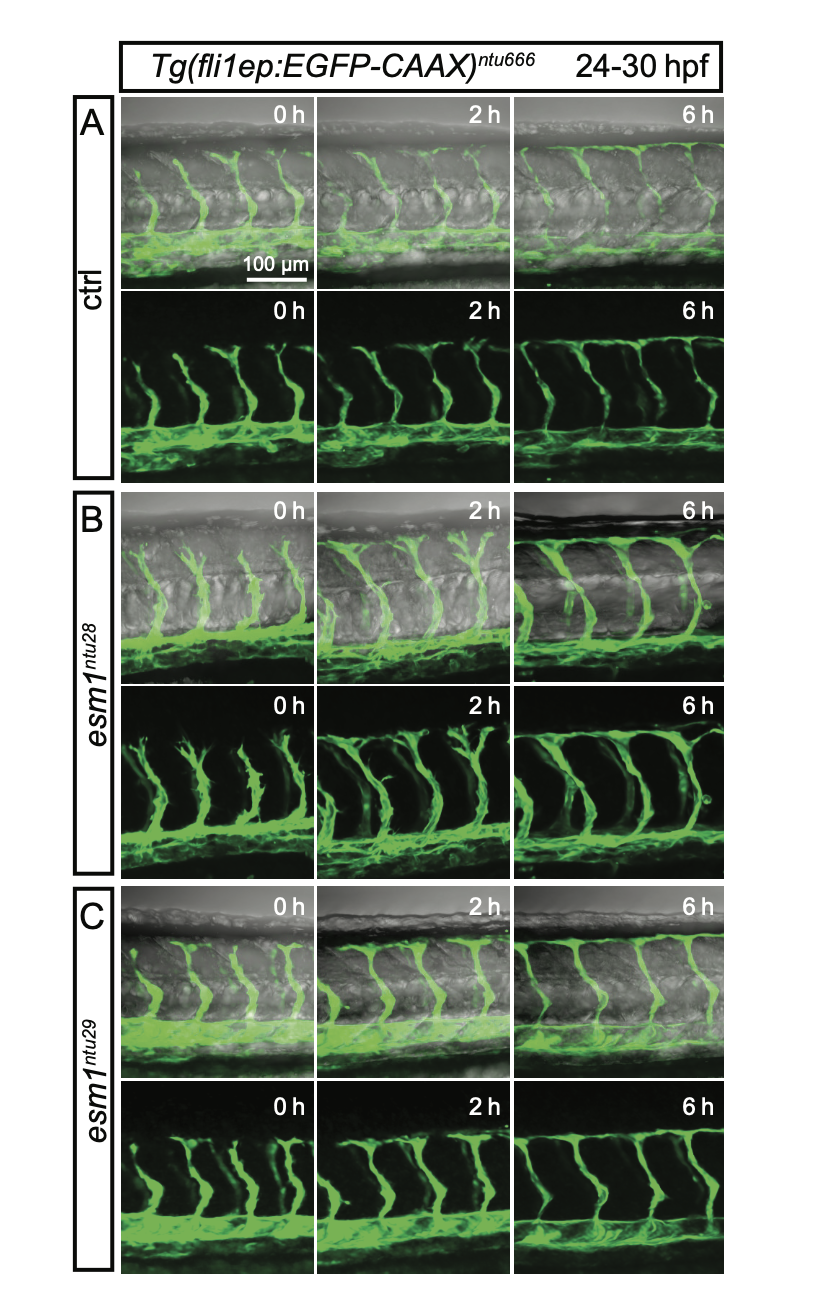


**Supplementary Figure 3. Still images from *in vivo* time-lapse imaging analysis of WT and *esm1*-knockout mutants from 24 to 30 hpf.**


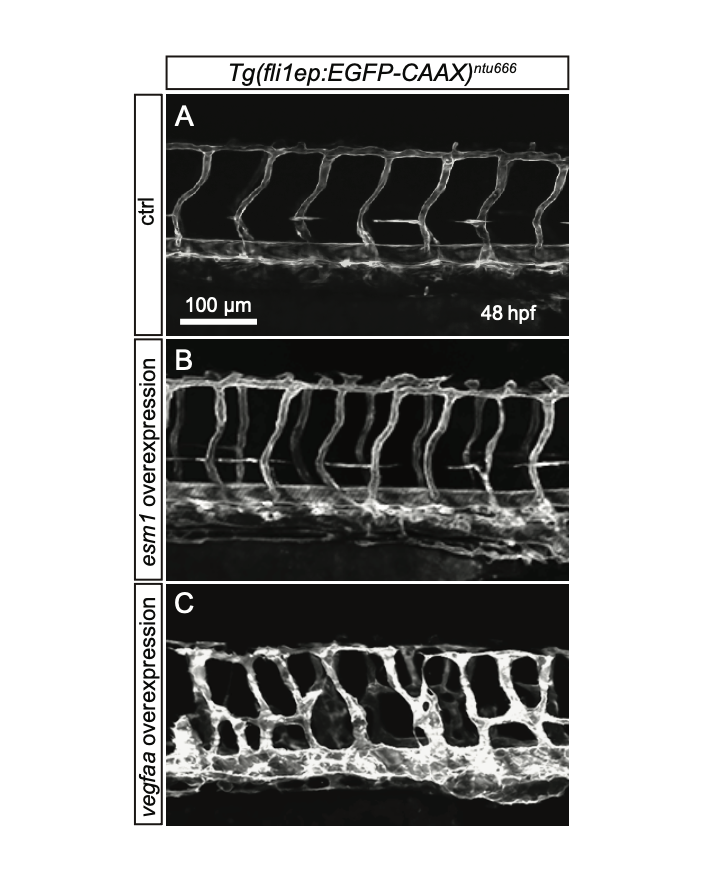


**Supplementary Figure 4. Comparisons of trunk vasculature in 48-hpf *Tg(fli1ep:EGFP-CAAX)^ntu666^* control embryos, and embryos overexpressing *esm1* or *vegfaa*.**


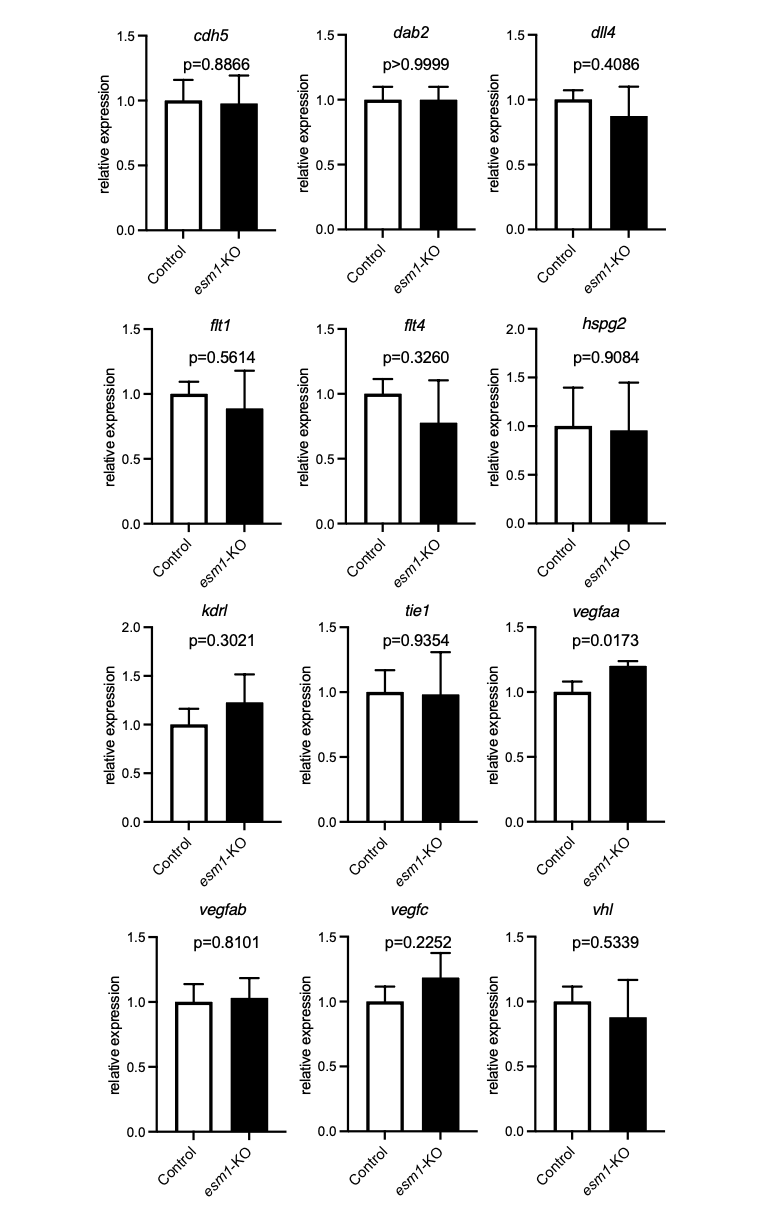


**Supplementary Figure 5. The expressions of angiogenesis- or vascular development-related genes in control embryos and *esm1^ntu28^* mutants.**
